# Supplementary material for: CMR Tissue Characterization in Patients with HFmrEF
Source: J Clin Med. 2019 Nov 5;8(11):1877. doi: 10.3390/jcm8111877 (PMC6912482; doi:10.3390/jcm8111877)
Supplement: Supplementary file 1 [file jcm-08-01877-s001.pdf]

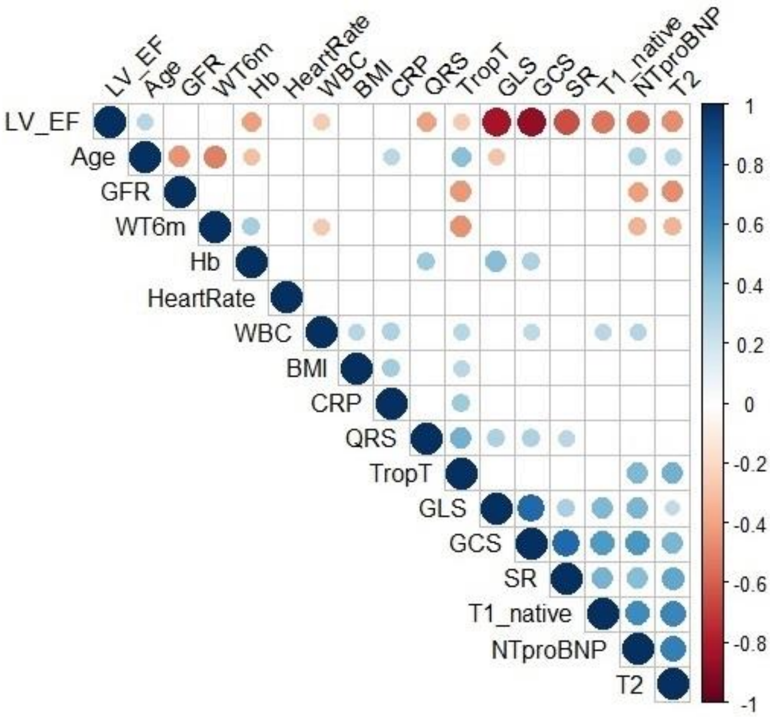

Figure S1

Correlation matrix for all continuous baseline and imaging parameters, created using the corrplot-library for R. [7] [12] NT-proBNP was transformed to logarithmic scale for correlation analysis. Positive correlations are displayed in blue and negative correlations in red color. Color intensity and the size of the circle are proportional to the correlation coefficients. Only significant (*P* value < 0.05) correlations are depicted. Abbreviations: WBC = White blood cell count, T2 = T2 relaxation time, logBNP = N-terminal pro brain natriuretic peptide (logarithmic scale), T1_native = Native T1 relaxation time, ECV = Extracellular volume, QRS = With of QRS complex, SR = Strain Ratio (GLS/GCS), GCS = Global circumferential strain, GLS = Global longitudinal strain, Hb = Hemoglobin, WT6m = 6 minute walking test, GFR = Glomerular filtration rate, LVEF = Left ventricular ejection fraction, BMI = Body mass index, CRP = C reactive protein, QoL = Quality of Life, TropT = Troponin T.

**Table S1.** Comparator Studies for Native T1[ms] and ECV[%]						
Native T1[ms]	System		Control	HFpEF	HFmrEF	HFrEF
Our study	1.5 T Philips		972±31	985 ±32	1027 ±40	1033 ±54
Rommel et al. 2016	1.5 T Philips		1,061 (1,000–1,097)	1,074 (1,044–1,107)		
Dabir et al. 2014	1.5 T Philips		950 ± 21			
Messroghli et al. 2006	1.5 T Philips		980 ± 53			
ECV[%]	System	Contrast	Control	HFpEF	HFmrEF	HFrEF
Our study	1.5 T Philips	0.15 mmol/kg gadobutrol		27.3 ±2.6	29.2 ±2.6	29.3 ±3.4
Roy et al. 2018	3 T Philips	0.2 mmol/kg gadobutrol	28.2 ± 2.4	32.9 ± 4.8		
Shelbert et al. 2017	1.5 T Siemens	0.2 mmol/kg gadobutrol		28.3 (25.5 to 31.4)		
Rommel et al. 2016	1.5 T Philips	0.15 mmol/kg gadobutrol	28.9 ± 3.0	32.9 ± 3.2		
Dabir et al. 2014	1.5 T Philips	0.15 mmol/kg gadobutrol	27 ± 4			
Su et al. 2014	3 T Siemens	0.15 mmol/kg gadobutrol	27.9 (26.2 to 29.4)	28.9 (27.8 to 31.3)		31.2 (29.0 to 34.1)

Data as Mean ± Standard Deviation or Median and Interquartile Ranges

Table S2. Differences in MRI-parameters by transmural LGE

Transmural LGE		N	Mean	SD	*P* value
T2	not present	27	55,0	4,7	0.599
	present	24	54,3	4,6	
ECV	not present	27	28,3	3,2	0.368
	present	23	29,1	2,7	
T1 native	not present	27	1015	47	0.865
	present	23	1017	49	

Table S3. Differences in MRI-parameters between females and males

		Control		HFpEF		HFmrEF		HFrEF		*P* value*
T2[ms]	Female	51,5	±1.6	53,0	±4.4	55,4	±3.5	58,0	±9.0	0.492
	Male	49,8	±2.2	52,1	±2.7	55,3	±3.5	55,6	±5.5	
ECV[%]	Female			27,0	±3.3	30,3	±2.0	30,7	±1.6	0.349
	Male			27,6	±1.9	28,7	±2.7	29,0	±3.7	
T1 Native[ms]	Female	986	±38	999	±26	1029	±35	1048	±98	0.251
	Male	959	±18	972	±34	1025	±45	1031	±45	
GLS[%]	Female	-23,4	±4.3	-22,9	±3.1	-15,8	±2.8	-8,7	±2.7	0.524
	Male	-22,6	±2.7	-18,5	±3.5	-15,7	±1.7	-11,5	±3.6	
GCS[%]	Female	-35,6	±5.9	-38,3	±7.6	-21,4	±3.6	-12,4	±7.8	0.176
	Male	-33,5	±6.7	-33,0	±4.4	-19,1	±4.3	-12,4	±4.1	
SR	Female	0,66	±0.10	0,61	±0.11	0,75	±0.14	0,882	±0.44	0.607
	Male	0,69	±0.08	0,56	±0.10	0,85	±0.18	0,979	±0.33	

* for difference between females and males
Data as Mean ± Standard Deviation or Median and interquartile Ranges
